# Recommendations for Diversifying Racial and Ethnic Representation in Autism Intervention Research: A Crossover Review of Recruitment and Retention Practices in Pediatric Mental Health

**DOI:** 10.3390/jcm11216468

**Published:** 2022-10-31

**Authors:** Wendy Machalicek, Lindsay Glugatch, Buket Erturk, Tasia Brafford, Megan Kunze, Christine Drew, Allaina Douglas, Sloan Storie, Rebecca Crowe, Sandy Magaña

**Affiliations:** 1 Department of Special Education and Clinical Sciences, College of Education, University of Oregon, Eugene, OR 97403, USA; 2 New Summit Behavioral Therapy, Springfield, OR 97478, USA; 3 Ordu Üniversitesi, Ordu 52200, Turkey; 4 Department of Special Education, College of Education, The University of Texas at Austin, Austin, TX 78712, USA; 5 Auburn University, Auburn, AL 36849, USA; 6 Instructional ABA Consultants, Inc., Naperville, IL 60563, USA; 7 University of Cincinnati, Cincinnati, OH 45221, USA; 8 Steve Hicks School of Social Work, The University of Texas at Austin, Austin, TX 78712, USA

**Keywords:** autism, literature review, minorities, recruitment, retention

## Abstract

Disparities in diagnosis and access to healthcare and therapeutic services are well-documented for children with autism spectrum disorder (ASD) from minoritized races and ethnicities, but there is little empirical research to guide the selection and implementation of interventions and practices that will effectively support racially/ethnically diverse children with ASD and their families. This cross-over systematic review summarizes parent-mediated intervention research of children with or at risk for mental health disorders to identify potentially effective recruitment and retention strategies for diverse participants in parent-mediated intervention research for children with autism. Electronic database keyword, lead author name searches in PyschNet, MEDLINE, and ancestral searches were conducted to identify 68 relevant articles that used experimental designs to evaluate the effects of parent-mediated interventions on children with or at risk for mental health disorders. Articles were coded for participant demographics; intervention setting and type, recruitment and retention strategies, cultural adaptation of intervention, and reported attrition. Findings are discussed and applied to practices in autism parent-mediated intervention research. Suggestions for future research and limitations are discussed.

## 1. Introduction

Disparities in diagnosis and access to healthcare and therapeutic services are well-documented for children with autism spectrum disorder (ASD) from minoritized races and ethnicities [1,2,3,4,5,6,7]. Moreover, there is little empirical research to guide the selection and implementation of interventions and practices that will effectively support racially/ethnically diverse children with ASD and their families [8,9,10]. Although the United States is increasingly a multicultural context, with Multiracial and Latinx being the fastest growing racial/ethnic categories in educational settings between 2011–2022, specifically children who identify as multiracial (44% increase) or Latinx (33% increase [11], only 21% of published ASD intervention research has reported inclusion of children from minorized racial/ethnic groups [12].

Recent research does present promising directions for intervention research with diverse families [13,14,15,16,17]. Magaña and colleagues [16,18,19] implemented a two-site randomized waitlist control group study (*n* = 96 mother-child dyads) examining the efficacy of a psychoeducation program delivered by *promotoras* (community health workers) as compared to usual services and support to improve Latina mother confidence in and use of evidence-based practices for children with ASD, decrease in ASD symptoms, and challenging behavior. Significant positive changes in maternal efficacy and frequency in the use of targeted evidence-based practices, child use of evidence-based services, and improved child social communication were observed in the treatment group. No significant differences were found in the treatment group family empowerment or child-challenging behavior for the overall sample, however, family empowerment significantly improved in the treatment group compared to the control in the California-based sample.

With a relative void of empirical studies reporting the inclusion of parent and child participants from diverse racial/ethnic groups, a first step may be to focus on effective recruitment, engagement, and retention of minoritized participants. Targeting these improvements makes sense as effective recruitment is essential for any intervention study with difficulties contributing to delayed protocol initiation, methodological adaptation, and study termination. Similarly, engagement and retention of participants for the duration of the intervention is necessary to maintain a sufficient sample size to determine treatment effects [20]. In RCTs, failure to complete the study protocol or attrition threatens trial validity and is linked to inflated treatment effect sizes [21]. In addition, from studies in clinical pediatric mental health, we know that early dropout from intervention has been correlated with lower socioeconomic status, child symptom severity, increased parenting stress, and minority racial/ethnic status [22,23]. The recruitment and retention of parents of children with a neurodevelopmental disorder such as ASD to parent-mediated intervention research may be especially challenging due to the time-intensive intervention. Moreover, recruitment of parents of children with ASD from minoritized races/ethnicities (e.g., Latinx) may be difficult due to cultural mistrust and experience with low-quality care [5].

A potential solution is for researchers to look to other populations, such as parent-mediated intervention for children with mental health concerns. The symptoms of ASD, common comorbidities, and familial stressors overlap with other mental health disorders, including attention deficit hyperactivity disorder (ADHD), obsessive compulsive disorder (OCD), oppositional defiance disorder (ODD), conduct disorder, externalizing challenging behavior, depression, and anxiety disorder [24]. Children with these diagnoses benefit from systematic instruction, and at times intensive intervention, including the involvement of multiple systems of care such as education and behavioral health [25]. The most commonly utilized and empirically supported practice to deliver content and skills to parents of children with the aforementioned concerns is behavioral parent training (BPT} [26,27,28]. The characteristics of this body of literature make it ideal for identifying potentially efficacious strategies to recruit and retain diverse participants in parenting intervention research focused on children with ASD.

### Purpose of the Present Study

The present study reports the findings of a systematic literature review of the parenting intervention research for parents of children with or at risk for mental health concerns to identify potentially efficacious recruitment and retention strategies for application to ASD parenting intervention research. A systematic literature review like this summarizes existing practices, discusses implications for practice, and recommends directions for future research. Diversity has been defined broadly as “…an individuals’ social identities including age, sexual orientation, [gender and gender identity], physical disability, socioeconomic status, race/ethnicity, workplace role/position, religious and spiritual orientation, and work/family concerns.” [29]. This paper focuses more narrowly on minoritized racial/ethnic status as reported in the childhood mental health intervention literature. We use the term “minoritized” rather than the term “minority” because nonwhite groups will soon no longer be the minority of the US population yet continue to be underrepresented in society’s institutions including research. The phrase, “racial and ethnic minoritized groups” better describes racial and ethnic groups that have less power and/or representation compared to other groups. We also use the phrase “racially and ethnically diverse” groups to depict minoritized racial and ethnic groups.

## 2. Methods

### 2.1. Design and Research Questions

This study followed a systematic narrative literature review design to exhaustively identify all published intervention study articles meeting our inclusion and exclusion criterion and to synthesize research methods across studies related to recruitment and retention practices. Since our review objectives did not include determining an overall estimate of the treatment effect of interventions, we did not report quality indicators of intervention research design and analysis but did ensure the articles contained sufficient information to inform our research questions pertaining to recruitment and retention. The following a priori research questions guided the present review of the literature:

1. What practices have researchers reported to recruit and retain ethnically/racially diverse participants in parenting models and programs targeting children with mental health disorders?
2. Are there opportunities for cross-fertilization from recruitment and retention strategies of racially and ethnically diverse populations used in parenting intervention research to address mental health disorders to improve the same practices in ASD intervention research?

### 2.2. Electronic Databases and Search Strategies

**Figure 1:**
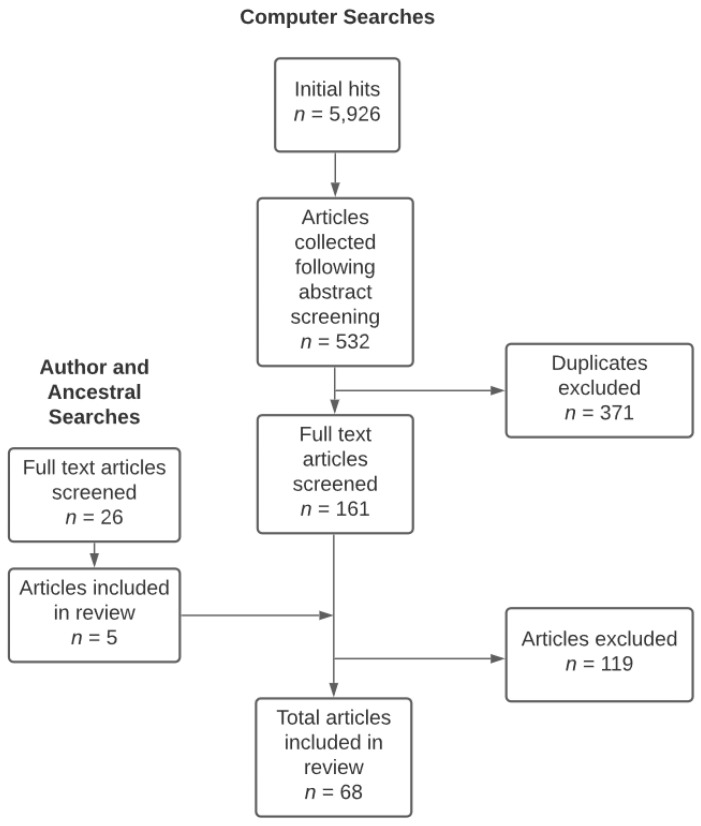
Search procedures used to identify articles for inclusion.

Articles were identified using a combination of electronic database searches using keywords and lead author name searches of authors appearing in included articles more than once and ancestral searches. Figure 1 illustrates the search procedures used to identify articles for inclusion in the present review.

Keyword searches were completed by authors using the electronic databases American Psychological Association PsychNET and MEDLINE by (a) entering one word or phrase from the following category one terms; (b) one word or phrase from category two terms; and (c) one word or phrase from category 3 terms. Category 1 terms included “parenting”, “parenting program”, or “intervention”. Category 2 terms included “disruptive beh*”, “emotional beh* dis*”, “externalizing beh*”, “ADHD”, “OCD”, “anxiety”, “social phobia”, “depression”, “conduct disorder”, or “oppositional defiance dis*”. Category 3 terms included “recruitment” and “adherence” or “minority”, “ethnicity”, “African American”, “Black”, “Hispanic”, “Latino”, “Latina”, “Pacific Islander”, “Native American” and “Alaskan”. Using these search terms, 161 articles were identified for the application of inclusion and exclusion criteria. 

### 2.3. Inclusion and Exclusion Selection Criteria

To be included in the review, articles (a) were published between 2011 and 2020. In 2011, the 2001 amended National Institutes of Health (NIH) Revitalization Act of 1993 [Public Law 103-43] mandating researchers to specify the appropriate representation and strategies for recruiting and retaining diverse participants, including women and non-dominant minorities had been in place for 10 years [30]; (b) written in English and conducted in the United States; (c) reported implementation of an operationally defined parenting model or training program in behavioral interventions in an outpatient, home, or school setting for at least one child between the ages of 3 and 18 years of age with or at risk for mental health disorders detailed in the search terms; and (d) reported an experimental or quasi-experimental design to evaluate the effects of the intervention on participant outcomes. After the removal of duplicates, the application of inclusion and exclusion criteria, and the ancestral review of the references and author name searches, 68 articles were included in the present review. Gray literature, literature reviews, and descriptive studies were not included in the analysis. A full list of the included articles is available as a supplemental file at 10.6084/m9.figshare.21433974.

### 2.4. Data Extraction and Coding

A 65-item Qualtrics survey developed by the authors was used to code each of the 68 articles on the following: participant and interventionist demographics; intervention setting, type, and characteristics; cultural adaptation of intervention; recruitment and retention strategies; and attrition rate. The authors piloted the survey with several practice articles and discussed coding discrepancies and subsequently revised the survey. Studies generally reported the proportion of child and parent participants meeting unique participant characteristics or demographic categories as ranges and proportions of the total study sample rather than the number of participants. Variance in the reporting of participant characteristics precluded reporting the true frequency of participants within each age group, race or ethnic category, gender, or diagnosis. We calculated participant data based on the proportion of reviewed studies that reported at least one participant meeting the specified characteristic/category. Included studies were classified into intervention categories according to the specific intervention described by the authors. The 5 categories were behavioral parent training (BPT), Parent-Child Interaction Therapy (PCIT), Cognitive Behavioral Therapy (CBT), Conjoint Behavioral Consultation (CBC), and Other. 

### 2.5. Interrater Reliability 

#### Article Inclusion and Coding

Two authors independently screened the full text of articles to apply inclusion and exclusion criteria to provide a measure of reliability on the inclusion of articles. Interobserver agreement (IOA) was calculated using the total agreement formula (Agreements/Agreements + Disagreements × 100). An IOA score of 86% was obtained. When authors disagreed about article inclusion, the authors discussed and came to a consensus about inclusion. 

To obtain an interrater reliability score for data extraction, a second author independently coded 40% or 27 of the 68 articles. IOA was calculated using the total agreement formula (Agreements/Agreements + Disagreements × 100) with each of the 65 survey items constituting a potential agreement or disagreement. An overall IOA score of 67% was obtained (1172 agreements/1755 items coded independently by two authors × 100). Lower agreement scores on nine items resulted from differences in how authors reported quantitative data (e.g., as the range of included age groups, rather than the mean age of participants). The coding sheet was updated following a discussion of discrepancies and a primary coder recoded those nine items for all 68 articles to ensure coding accuracy and uniformity on these items across the reviewed articles, which was verified by the second and third authors.

## 3. Results

### 3.1. Participant Characteristics

Across the 68 studies, 41 (60%) evaluated intervention with one or more of the following groups: minoritized racial/ethnic groups, families living in poverty, and underserved families. Sixteen studies reported inclusion of families living in poverty [31,32,33,34,35,36,37,38,39,40,41,42,43,44,45,46], or underserved families [38,47,48,49,50,51].

A smaller number of studies (*n* = 10 studies; 15%) reported a participant sample with more than 50% of participants identifying as Hispanic or Latinx [34,35,42,43,46,52,53,54,55,56]. Four of these studies recruited Hispanic or Latinx families from low SES backgrounds [34,35,43,46]. McCabe et al. included Mexican-American families [57]. Few studies (*n* = 5) targeted recruitment of Black/African American parents [33,38,42,52,58] and one study reported immigrant Chinese participants [59]. Of the 20 studies not reporting targeting a specific minoritized population, all but a small minority (*n* = 4) reported recruitment and/or retention strategies.

#### 3.1.1. Children

Across the 68 reviewed studies, 9114 children met our inclusion criteria. The majority of studies (87%; *n* = 59 studies) reported the inclusion of at least one child between birth and eight years of age and 76% (*n* = 52 studies) reported the inclusion of at least one child between 9 and 12 years of age. Children over 13 years of age were reported in 38% of studies. Age was not reported in two studies. Over half of the studies reported at least one white, Black, or Latinx participant (66%, 65%, and 59% of studies, respectively), and 40% of studies reported at least one multi-racial participant. A smaller number of studies reported at least one participant who was Native American (16% of studies), Asian (16% of studies), or Pacific Islander (6%). Half of the studies (*n* = 32) reported at least one child participant with ADHD who engaged in externalizing challenging behavior (*n* = 19; 28% of all studies) or disruptive conduct (*n* = 14; 21% of all studies); however, 16% of studies reported at least one child participant with an internalizing diagnosis such as an anxiety disorder or social phobia. However, 18% (*n* = 12 studies) of studies failed to report participant diagnosis or identified the participants as at-risk for diagnosis (*n* = 13; 19%), which reflects the large proportion of studies focused on preventative interventions for at-risk children and parents.

#### 3.1.2. Parents

A majority of studies (*n* = 36; 53%) identified parents or caregivers as mothers, though fathers were identified as caregiver participants in 28% of all studies. Caregiver participants’ genders were not identified in 49% of studies. When reported, participating parents reported varying educational experiences between less than a high school diploma to doctorate level, but 49% of studies did not report parent education level. Socio-economic status tended towards a $50,000 annual income or less (*n* = 32 of 68 studies), and 18 studies reported family incomes of less than $15,000. The most commonly reported income category was $15,000 to $49,999. Twenty-two studies did not report income, and eight studies reported family incomes of $75,000 or higher. Table 1 summarizes participant demographics across the included studies.

### 3.2. Intervention Settings and Types of Intervention

Fewer than one-half of the studies (44% of studies) reported the geographical setting of the intervention. When reported, the majority of interventions were implemented in Urban settings (37% of studies) or both Urban and Rural settings (7%). More information was reported about the specific intervention settings with 50% of intervention studies implemented in community settings, 15% of studies implemented in school settings, and 19% of intervention studies carried out in both school and community settings. Only 18% of studies failed to report this information.

### 3.3. Parenting Programs, Models, and Delivery Format

Twenty-one percent (*n* = 14) of studies reported delivering intervention content using a general behavioral parent training (BPT) approach. BPT is characterized by the combined use of parent education with modeling, role play, and coaching with performance feedback while the parent practices skills with their child. Parent-Child Interaction Therapy (PCIT), which includes BPT, was examined by 11 studies (16%) and cognitive behavioral therapy by six studies (9%). Conjoint behavioral therapy, which includes BPT, across home and school settings was evaluated by three studies (4%). The remainder of the studies either examined unique manualized interventions (e.g., Parent Management Training-Oregon Model; Partnering to Achieve Student Success; Preschool PATHS Curriculum; Parenting Our Children to Excellence; Cool Kids Face to Face; Chicago Parent Program; Culturally Informed and Flexible Family-Based Treatment for Adolescents; The Kids in Transition to School Program; or non-manualized intervention approaches such as eclectic psychotherapy, exposure response prevention (ERP) therapy, or culturally enhanced video feedback engagement.

Interventions were far more likely to be delivered in a face-to-face format (*n* = 56 studies) than online (*n* = 3 studies) or using a hybrid approach of delivery with both face-to-face and online components (*n* = 3 studies). Six studies did not report intervention modality. Researchers were more likely to report individual (41% of studies) parent training than either group training (24% of studies) or both individual and group training (25%). Some studies (6% of studies) did not report this information.

### 3.4. Recruitment and Retention Strategies

Table 2 reports the number of studies reviewed reporting specific recruitment and retention strategies.

Across the 59 studies reporting recruitment strategies, a variety of recruitment strategies were represented in the literature and are defined in Table 3 with exemplar studies cited. 56% of those studies targeting recruitment of racially/ethnically diverse families reported cultural adaptation of the intervention. For the 23 studies utilizing a single recruitment strategy, recruitment strategies from most to least frequently employed were school-based recruitment (*n* = 10 studies), face to face recruitment at community providers (*n* = 8 studies), direct referrals (*n* = 4 studies), and self-referral (*n* = 1 study). Recruitment strategies were more commonly deployed in combination (*n* = 36 studies; 53%). For Latinx/Hispanic or Mexican American families, researchers use school-based recruitment, direct referrals, community agency, radio/TV advertisement or magazine or newspaper advertisements, flyers, and self-referral. For the recruitment of Black families, one study used school-based recruitment only, one study relied on self-referrals and direct referrals from mental health providers, and another study used a combination of referrals from school counselors and community providers. The study recruiting Chinese immigrant families used school-based recruitment, direct referrals, and community agencies. For researchers recruiting low SES families with multiple methods, the most frequently used strategy was school-based, followed closely by direct referrals and community agencies. Flyers, community setting, self-referral, and Internet/social media were also used. Due to the relatively low numbers of studies targeting specific populations and the diversity in recruitment strategies used across studies, we discerned no pattern in the use of multiple recruitment strategies. Participant self-referral was utilized in 15 studies. 

Similarly, across the 33 studies reporting retention strategies, a number of strategies were utilized by researchers in an effort to retain participants during the entirety of the intervention study (see Table 4) [35,37,39,49,60,61]. These strategies included providing parents with a financial stipend for participation (*n* = 21 studies) or gift cards (*n* = 6 studies); allowing for flexible scheduling of intervention sessions (*n* = 10 studies), providing the parent with a choice of intervention (*n* = 1), providing childcare (*n* = 7 studies) or items thought to reinforce participation, such as small toys for the children (*n* = 1 study) or food (*n* = 4 studies). Other strategies included the provision of educational materials (*n* = 6 studies), the use of reminders to engage in intervention activities (*n* = 7 studies), and motivational interviewing (*n* = 2 studies). Seventeen studies used two or more retention strategies. Researchers recruiting low-income and urban families reported retention strategies in 65% and 61% of these studies, respectively. Researchers recruiting underserved families (40%), Latino/Hispanic families (35%), and Black/African American families (24%) also used retention strategies and it appears that researchers recruiting specific populations (urban not included) were more likely to use multiple retention strategies.

### 3.5. Cultural Adaptation

A number of studies (*n* = 27 studies) adapted intervention procedures for specific populations. One-half of the studies targeting Hispanic/Latinx families or Black/African American Families reported intervention adaptation [42,43,46,52,53,55,58]. Those studies targeting Chinese immigrants and Mexican-Americans also reported cultural adaptation [57,59]. Additionally, 54% of the studies targeting low SES families reported some adaptation of the intervention [32,33,36,37,39,41,42]. The most common way in which the researchers adapted the intervention included increased accessibility/reduced effort (*n* = 14), the intervention provided by a bilingual interventionist (*n* = 9), use of culturally relevant themes within the intervention (*n* = 8), involving stakeholders in the development of the intervention (*n* = 7), and translating materials (*n* = 6). Some researchers used a combination of these procedures.

### 3.6. Attrition

The reasons for attrition were often not reported in studies (*n* = 48 studies), but we can ascertain from reported data that 4395 participants completed the study, and 510 participants dropped out. Table 5 provides a summary of reported reasons for participant drop out and disconnection [32,37,38,48,53,62,63,64,65].

## 4. Discussion

This review aimed to summarize practices to recruit and retain minoritized racial and ethnic groups in parenting intervention targeting children with or at risk for mental health concerns, and apply the results to identify promising strategies for increasing participant racial/ethnic diversity in parent-mediated intervention research with individuals with ASD. An array of recruitment and retention strategies have been combined by researchers to meet perceived participant needs and mitigate barriers to participation and a number of studies have described culturally adapted interventions to address the support and intervention needs of specific racial/ethnic populations. Our findings and suggestions for future research and practice are discussed in relation to our two a priori research questions.

### 4.1. Practices Reported by Researchers to Recruit and Retain Minoritized Participants

Researchers in pediatric mental health have used a variety of strategies to recruit and retain ethnically/racially diverse parents and children. These practices have aimed to (a) increase access to information about studies (e.g., distribution of study information in flyers to potentially interested communities, information about the study in languages other than English, bilingual research personnel), and (b) overcome participation barriers through the use of flexible appointment scheduling and financially supporting transportation and childcare [66]. Researchers commonly combined partnering with community agencies or schools already serving children and their families, and providing tokens of appreciation to families for study participation (e.g., gift cards). These strategies are reported in the public health intervention literature [67] and have been used to recruit specific racial/ethnic groups in behavioral research [68]. Future research should determine which strategies are causally associated with enrollment time, sample diversity (i.e., the proportion of participants from non-white racial/ethnic groups), and participant engagement. Zamora and colleagues [69] provide a model of experimental evaluation of the effects of culturally-specific strategies as compared to traditional strategies on engaging Latino families of children with ASD in genetic research. Although traditional recruitment methods (dual language flyers, clinician invitation) were effective in recruiting both Latino and non-Latino families, Latino families were more responsive to the culturally relevant recruitment strategies of communication from a community-based organization partnering with the researchers and invitation from a parent who had already participated in the study [69]. These findings suggest the utility of partnering with schools and community-based organizations who already have already earned the trust of families and hiring past participants as recruitment specialists. 

This review found that researchers more often utilize multiple recruitment strategies, rather than single strategies in isolation. More than half of the studies reported the use of two or more recruitment strategies, including some researchers combining a large number of strategies. For example, rather than just providing vouchers to assist participants in traveling to the assessment site, researchers combined vouchers with onsite childcare during assessment appointments, flexible appointment scheduling, and adaptation of the intervention to reduce participant effort and time. The provision of multiple strategies is sensible, considering the overlapping barriers that recruitment and retention strategies seek to remedy. For instance, the lack of childcare and reliable transportation during study visits are often both experienced by families; offering a solution to mitigate one barrier but not another may be ineffective. Although the use of multiple strategies is logical, the absence of experimental research on the effects of the strategies individually or as a package necessitates a deploy and hope method. Experimental demonstrations of the effects of specific recruitment and retention packages on sample size and diversity, as well as participant engagement and attrition rates, would be helpful. Additionally, qualitative research reporting perceptions of participants regarding the goals, procedures, and outcomes of intervention research could assist in identifying which recruitment and retention strategies research participants found most helpful in recruiting them to the study and maintaining their engagement in the intervention.

### 4.2. Opportunities for Recruitment and Retention in ASD Parenting Intervention Research

The recruitment and retention practices reported in this review have an important role in ensuring culturally responsive and effective recruitment and retention in autism research [9]. Extant research studies involving minoritized families of children with autism have used a multi-pronged approach to recruitment and retention similar to that found in this review. For instance, Carr & Lord [14] piloted an adapted version of the Early Social Interaction Project (ESI) [70] with eight low-resourced, English-speaking families of young children with ASD using a one-group pre-test post-test design. Adaptations included modified location (family home, rather than clinic), transportation support (i.e., taxi service or travel funds), eligible age range, decreased duration and frequency of intervention, liberal cancellation, and rescheduling procedures, monetary incentive for completed assessments, and case management assistance to obtain ASD services beyond the research study. However, this approach requires more wide-spread use to document the effectiveness of recruitment and retention strategies for this population. In Carr & Lord [14], five families completed the study, and attendance varied from 3.6 to 9.1 months due to family cancellation of intervention sessions.

There are several areas where researchers can improve practices including (a) reporting requirements for recruitment and retention strategies; (b) the development of interventions that address population-relevant issues; (c) the development of relational and culturally relevant approaches; and (d) minimizing barriers to engagement in intervention research.

#### 4.2.1. Reporting Requirements for Recruitment and Retention Strategies

The strategies discussed in this review are relatively straightforward to implement and may already be in use (but not reported) by researchers studying parent-implemented interventions for children with ASD. To allow for examination of the effectiveness of these strategies with children with ASD and their parents, we recommend researchers report the racial/ethnic backgrounds of participants and any recruitment and retention efforts deployed, and editors require this information in the published article or as a supplemental file. Similarly, we encourage researchers to report measures of participant engagement in the intervention, attrition and reported reasons for dropouts.

#### 4.2.2. Develop Interventions That Address Population Relevant Issues

To create interventions with an enhanced contextual fit, researchers may consider community-based participatory research methods [71] or diffusion of innovation framework [9]. Community-participatory research has been defined as leadership by researchers with autism, partnership with people with autism or allies to co-create the intervention, community engagement, and consultation with community organizations [72,73,74]. Well-described exemplars of researchers partnering with schools to adapt existing interventions do exist and provide models for researchers seeking to systematically involve stakeholders, including parents of children with autism and adults with autism in the development of interventions [75,76].

We recommend researchers develop and evaluate the effectiveness of recruitment and retention practices specifically targeting malleable variables associated with attrition in BPT and behavioral health treatment more generally, such as parental stress and depression, and co-occurring psychiatric and medical conditions, including child sleep disorders [14,77]. For instance, parents of children with autism present with higher rates of elevated stress and depression when compared to parents of children with other developmental disabilities [78,79]. Relatedly, poorer outcomes for children participating in intensive behavioral intervention have been associated with higher levels of parent stress [80]. A potential area of further research is to develop and test engagement strategies for parents of children with autism that might address heightened stress. Such strategies might include social and emotional support and assistance in connecting with other families among other supportive strategies [81].

#### 4.2.3. Relational and Culturally Relevant Approaches to Intervention Development

Researchers seeking to engage minoritized families can also develop culturally situated interventions that involve stakeholders in intervention development, use bicultural, bilingual intervention agents, translate intervention materials, and incorporate culturally relevant themes. The incorporation of culturally relevant themes, such as *personalismo* and *respeto* for Latinx families, and addressing the racial contexts experienced by Black families, including experiences of racism [82,83], may be of greater utility than recruitment and retention efforts alone. A first priority should be to improve the inclusion of diverse families into existing intervention trials, but selective and directed intervention adaptation addressing known issues affecting intervention participation may be an efficient way to approach cultural adaptation [84]. Additionally, some culturally relevant themes universally put into practice may serve the needs of families from a variety of backgrounds when combined with individualized problem-solving [85]. For instance, research supports the use of parent-to-parent support between a parent who is new to their child’s diagnosis of a mental health concern and a parent who has already successfully navigated educational and mental health systems to support parents in mitigating barriers to receipt of intervention and other services [86]. Based on this review, it appears that those strategies which are helpful in recruiting minoritized racial/ethnic families may also increase socio-economic diversity in research samples as a large number of participants in this review were considered financially under-resourced. 

Relationship-centered approaches including the use of family partner advocates, family peer support partners, or *promotoras* in Latinx research, have demonstrated effectiveness in mental health [87] and autism intervention research with Black and Hispanic/Latinx parents [16,88]. Multiple family group intervention (MFG) has also been used successfully with parents of school-age children with disruptive behavior disorders to allow for efficient mental health service provision to multiple families from disadvantaged backgrounds at one time while providing a source of support from other parents experiencing similar challenges [86,89]. Group-based parent interventions also have the advantage of serving multiple families and potentially easing access to the intervention. These relational approaches may be more acceptable to ethnically/racially diverse families while offering much-needed social support and validation of family experiences [86]. 

#### 4.2.4. Identify and Minimize Barriers to Engagement in Intervention Research

Researchers can remove known barriers associated with non-engagement in research by explicitly inviting non-English speaking and other minority groups to participate in research [90], planning for use of research sites that are accessible and acceptable to minority groups, and by providing childcare and travel support [91]. Clinical trials often require research participants to be fluent in English, in part due to the lack of standardized assessments and intervention procedures in other, non-dominant languages [5], so translation into languages other than English is essential.

Additionally, for families who have recently emigrated to their current country of residence, recruitment and retention strategies may benefit from adaptation to address the considerable cultural stressors (stigma, acculturation) families face due to their non-dominant status in society [92], which may impact engagement in parent-mediated intervention. For instance, a heightened level of stress is associated with depressive symptoms for Latinx parents, and both stress and depression have been linked to poorer family functioning and adolescent well-being, and health risk-taking behavior [93]. Parents who have recently emigrated and are financially insecure parents may benefit from assistance obtaining resources addressing financial instability, housing, education, and transportation in combination with child-centered services to prevent worsened longitudinal outcomes. Moreover, the social, political, and economic context of the community and society at the time of the study may impact participation in research. For example, punitive immigration policies negatively impact the efforts of researchers to recruit immigrants to participate in health behavior research due to justifiable fears of deportation [94]. Researchers must first and foremost maintain awareness of the political climate and the stressors immigrant families who have children with ASD face. Additionally, researchers may wish to consider providing additional resources and service navigation to immigrant families to reduce attrition.

### 4.3. Limitations of the Review

Relatively few studies experimentally examined the effects of recruitment and retention efforts, few reported attrition rates, and reported outcomes were heterogeneous. These findings prevented an analysis of the effectiveness of the strategies on attrition and engagement. This paper focused on ethnicity/race and SES status. Researchers are cautioned to purposefully recruit participants that belong to multiple minoritized categories to reflect true diversity [95]. Researchers should aim for intersectionality in their sample to include various gender identities, immigration statuses, and social classes [95,96,97]. Finally, this manuscript did not discuss contextual and structural barriers related to the recruitment of minoritized families to research studies. The social, political, and economic context of the community and society at the time of the study may impact participation in research. We urge researchers to consider the findings of this paper within the larger socio-cultural climate at the time of recruitment.

## 5. Conclusions

The continued absence of minoritized racial/ethnic groups in ASD intervention research is a health disparity issue related to which populations benefit from research, how decisions about research and intervention are made, and the types of questions asked about the intervention targets and diverse populations [97]. We urge researchers to use the findings of this review to improve the inclusion of diverse children with autism and their families in intervention research to (a) better distribute the benefits and risks of research participation, (b) examine research questions related to the feasibility, acceptability, and effectiveness of intervention programs or models with diverse populations, and (c) explore potential variables associated with specific populations that interact with intervention effects or participation in research; these scholarly avenues are well-defined in the clinical health literature [98].

## Figures and Tables

**Table 1:** Participant Demographics Across Research Studies.

Demographic Characteristic	*n*	Percentage of Studies
Participant age		
Birth—2 years old	6	18%
3–6 years old	24	73%
7 years old and older	25	76%
Ethnicity		
White	24	73%
Black or African American	19	58%
Hispanic or Latino/a	17	52%
Bi- or multi-racial	13	39%
Other	8	24%
Native American or American Indian	6	18%
Asian	5	15%
Non-Hispanic	4	12%
Not reported	2	6%
Non-white	1	3%
Socioeconomic status		
Indicators of low- or under-resourced families	20	61%
Not reported or cannot be determined	11	33%

Note. Percentage of studies indicates the ratio of studies reporting at least one participant in the specific demographic category in relation to the total number of studies (*n* = 33). Low- or under-resourced status determined by authors’ description or an income of $20,000 or below.

**Table 2:** Number of Studies Reporting Specific Recruitment and Retention Strategies.

		Retention Strategies					
Recruitment Strategies	Money or Gift Cards	Free Services or Childcare	Educational Materials	Flexible Scheduling	Reminders	Choice of Intervention	Not Reported
School	8	3	2	1	1		4
Direct referral	3	3	1	1	1	1	8
Community agencies or setting	5	3	1	1	2		5
Physicians or service providers	4	3	1	1	1		5
Flyers or mailings	6	1	2	1			5
Workshops or open house	2						1
Self-referral	1	2					4
Trusted person	2	1	1				1
Word-of-mouth	3		1	1			
Radio or newspaper	2						1
University clinic or faculty		1			1		1
Online or social media	2						
Child Protective Services		1					
Not reported	1	1	1	2			2

Note. Cells include the number of studies reporting the corresponding retention and recruitment strategies.

**Table 3:** Reported Reasons for Drop-out or Disconnection Across Studies.

Reason	Study *n*	Participant *n*
Not reported	18	394 (77%)
Withdrew consent	5	11 (2%)
Relocation	6	35 (7%)
Lost or could not contact (e.g., disconnected phone, unreturned communication attempts)	3	35 (7%)
Medication or medical conflicts	3	11 (2%)
Transportation	2	3 (1%)
Family reasons	1	3 (1%)
Perceived lack of need	1	11 (2%)
Teacher refused	1	3 (1%)

Note. Reviewed studies either did not report reasons, had all participants complete their respective studies, or reported one or more reasons participants withdrew from study. Number of participants completed (*n* = 4395). The number of participants who dropped out (*n* = 510) was used to determine the percentage of each drop out reason.

**Table 4:** Recruitment Strategies Across Research Studies.

Recruitment Strategy	Definition	Exemplar Article
School referral	School personnel involved in recruitment (referral or direct involvement	60
Community agency	Community agency (physician, early childhood provided, mental health, etc.) involved in recruitment (referral or other)	39
Direct referral	Community provider directly refers a participant to the researcher	35
Self-referral	Families contact the researcher directly	61
Flyers	Posted, mailed, e-mailed, or handed out in person	49
Community setting/outreach	Face-to-face outreach in community settings that provide leisure or recreation services	37
Radio or TV advertisements	Advertisements created for radio or television mediums	35

Note. Some reviewed articles did not report, did not engage in, or reported multiple recruitment strategies. Definitions were created using exemplar studies.

**Table 5:** Retention Strategies Across Research Studies.

Retention Strategy	Exemplar Study	Studies *n*
Money	62	19
Flexible scheduling	63	8
Childcare	53	6
Gift card	64	6
Reminder	48	6
Educational materials	37	6
Food	32	3
Motivational interviewing Choice of intervention	65 38	1 1

Note. Some reviewed articles did not report, did not engage in, or reported multiple retention strategies. Definitions were created using exemplar studies.

## Data Availability

Data extracted from articles were collected and analyzed using Qualtrics and excel. Resultant data is available by request.

## Supplementary Materials

The following supporting information can be downloaded at: https://www.mdpi.com/article/10.3390/jcm11216468/s1 or www.figshare.com, A list of articles included in the review 10.6084/m9.figshare.21433974.
