# Supplementary material for: Recommendations for Diversifying Racial and Ethnic Representation in Autism Intervention Research: A Crossover Review of Recruitment and Retention Practices in Pediatric Mental Health
Source: J Clin Med. 2022 Oct 31;11(21):6468. doi: 10.3390/jcm11216468 (PMC9654487; doi:10.3390/jcm11216468)
Supplement: Supplementary file 1 [file jcm-11-06468-s001.zip › jcm-1973091-supplementary.pdf]

Supplemental File S1

Articles included in the literature review

1. Antshel, K. M., Faraone, S. V., & Gordon, M. (2014). Cognitive behavioral treatment outcomes in adolescent ADHD. *Journal of Attention Disorders*, *18*, 483-495.

2. Bagner, D. M. (2013). Father's role in parent training for children with developmental delay. *Journal of Family Psychology*, *27*, 650-657. doi:10.1037/a0033465.
3. Baker, C. N., Arnold, D. H., & Meagher, S. (2011). Enrollment and attendance in a parent training prevention program for conduct problems. *Prevention Science*, *12*, 126-138. doi:10.1007= s11121-010-0187-0
4. Bauer, N.S., Szczepaniak, D., Sullivan, P.D., Mooneyham, G. Pottenger, A., Johnson, C.S., & Downs, S. M. (2015). Group visits to improve pediatric attention-deficit hyperactivity disorder chronic care management. *Journal of Developmental and Behavioral Pediatrics*, *36*(8), 533- 561.

5. Bierman, K. L., Nix, R. L., Heinrichs, B. S., Domitrovich, C. E., Gest, S. D., Welsh, J. A., & Gill, S. (2014). Effects of Head Start REDI on children's outcomes 1 year later in different kindergarten contexts. *Child Development*, *85*(1), 140-159. https://doi.org/10.1111/cdev.12117
6. Breitenstein, S. M., Gross, D., Fogg, L., Ridge, A., Garvey, C., Julion, W., & Tucker, S. (2012). The Chicago Parent Program: Comparing 1-year outcomes for African American and Latino parents of young children. *Research in Nursing & Health*, *35*(5), 475-489. https://doi.org/10.1002/nur.21489

7. Carrion, V. G., Kletter, H., Weems, C. F., Berry, R. R., & Rettger, J. P. (2013). Cue-centered treatment for youth exposed to interpersonal violence: A randomized controlled trial. *Journal of Traumatic Stress*, *26*(2), 654-662. https://doi.org/10.1002/jts.21870

8. Chacko, A., Gopalan, G., Franco, L., Dean-Assaei, K., Jackson, J., Marcus, S., Hoagwood, K., & McKay, M. (2015). Multiple family group service model for children with disruptive behavior disorders: Child outcomes at post-treatment. *Journal of Emotional and Behavioral Disorders*, *23*(2), 67-77. https://doi.org/10.1177/1063426614532690
9. Chavira, D. A., Drahota, A., Garland, A. F., Roesch, S., Garcia, M., & Stein, M. B. (2014). Feasibility of two modes of treatment delivery for child anxiety in primary care. *Behaviour Research & Therapy*, *60*, 60-66.
10. Chronis-Tuscano, A., O'Brien, K. A., Johnston, C., Jones, H. A., Clarke, T. L., Raggi, V. L., Rooney, M. E., Diaz, Y., Pian, J., & Seymour, K. E. (2011). The relation between maternal ADHD symptoms & improvement in child behavior following brief behavioral parent training is mediated by change in negative parenting. *Journal of Abnormal Child Psychology*, *39*, 1047-1057. https://doi.org/10.1007/s10802-011-9518-2

11. Chronis-Tuscano, A., Rubin, K. H., O'brien, K. A., Coplan, R. J., Thomas, S. R., Dougherty, L. R., ... & Menzer, M. (2015). Preliminary evaluation of a multimodal early intervention program for behaviorally inhibited preschoolers. *Journal of Consulting and Clinical Psychology*, *83*(3), 534-40. doi: 10.1037/a0039043

12. Clarke, A. T., Marshall, S. A., Mautone, J. A., Soffer, S. L., Jones, H. A., Costigan, T. E., ... & Power, T. J. (2015). Parent attendance and homework adherence predict response to a family–school intervention for children with ADHD. *Journal of Clinical Child & Adolescent Psychology*, *44*(1), 58-67.
13. Conduct Problems Prevention Research Group. (2011). The effects of the Fast Track preventive intervention on the development of conduct disorder across childhood. *Child Development*, *82*(1), 331-345. doi: 10.1111/j.1467-8624.2010.01558.x
14. Danko, C. M., Garbacz, L. L., & Budd, K. S. (2016). Outcomes of Parent–Child Interaction Therapy in an urban community clinic: A comparison of treatment completers and dropouts. *Children and Youth Services Review*, *60*, 42-51.

15. Dawson-McClure, S., Calzada, E., Huang, K.-Y., Kamboukos, D., Rhule, D., Kolawole, B., Petkova, E., & Miller Brotman, L. (2015). A population-level approach to promoting healthy child development and school success in low-income, urban neighborhoods: Impact on parenting and child conduct problems. *Prevention Science*, *16*, 279-290. https://doi.org/10.1007/s11121-014-0473-3
16. Dawson, A. E., Wymbs, B. T., Marshall, S. A., Mautone, J. A., & Power, T. J. (2016). The role of parental ADHD in sustaining the effects of a family-school intervention for ADHD. *Journal of Clinical Child & Adolescent Psychology*, *45*(3), 305-319.
17. Dumas, J. E., Arriaga, X. B., Begle, A., M., & Longoria, Z. N. (2011). Child and parental outcomes of a group parenting intervention for Latino families: A pilot study of the CANNE Program. *Cultural Diversity and Ethnic Minority Psychology*, *17*, 107-115. https://doi.og/10.1037/a0021972
18. Eckshtain, D., Kuppens, S., & Weisz, J. R. (2017). Amelioration of child depression through behavioral parent training: A preliminary study. *Journal of Clinical Child and Adolescent Psychology*, *46*(4), 611-618. https://doi.org/10.1080/15374416.2015.1050722
19. Esposito-Smythers, C., Spirito, A., Kahler, C. W., Hunt, J., & Monti, P. (2011). Treatment of co-occurring substance abuse and suicidality among adolescents: A randomized trial. *J. Consult. Clin. Psychol.*, *79*, 728-739.
20. Fernandez, M. A., Butler, A. M., & Eyberg, S. M. (2011). Treatment outcome for low socioeconomic status African American families in parent-child interaction therapy: A pilot study. *Child & Family Behavior Therapy*, *33*(1), 32–48. https://doi.org/10.1080/07317107.2011.545011
21. Ginsburg, G. S., Becker, K. D., Drazdowski, T. K., & Tein, J. Y. (2012). Treating anxiety disorders in inner city schools: Results from a pilot randomized controlled trial comparing CBT and usual care. In *Child & Youth Care Forum*, *41* (1), 1-19.
22. Gomez, D., Bridges, A. J., Andrews, A. R., Cavell, T. A., Pastrana, F. A., Gregus, S. J., & Ojeda, C. A. (2014). Delivering parent management training in an integrated primary care setting: Description and preliminary outcome data. *Cognitive and Behavioral Practice*, *21*(3), 296-309.
23. Graziano, P., Slavec, J., Hart, K., Garcia, A., & Pelham, W. (2014). Improving school readiness inpreschoolers with behavior problems: Results from a summer treatment program. *Journal of Psychopathology and Behavioral Assessment*, *36*, 555-569. https://doi.org/10.1007/s10862-014-9418-1
24. Graziano, P. A., Bagner, D. M., Slavec, J., Hungerford, G., Kent, K., Babinski, D., Derefinko, K., & Pasalich, D. (2015). Feasibility of intensive parent–child interaction therapy (IPCIT): Results from an open trial. *Journal of Psychopathology and Behavioral Assessment*, 37(1), 38–49. https://doi.org/10.1007/s10862-014-9435-0
25. Graziano, P. A., & Hart, K. (2016). Beyond behavior modification: Benefits of social emotional/self-regulation training for preschoolers with behavior problems. *Journal of School Psychology*, *58*(2016), 91-111. https://doi.org/10.1016/j.jsp.2016.07.004
26. Gross, D. A., Belcher, H. M.D., Ofonedu, M. E., Breitenstein, S., Frick, K. D., & Chakra, B. (2014). Study protocol for a comparative effectiveness trial of two parent training programs in afee-for-service mental health clinic: Can we improve mental health services to low income families? *Trials*, *15*(70), 1-10. https://www.trialsjournal.com/content/15/1/70
27. Hart, K. C., Graziano, P. A., Kent, K. M., Kuriyan, A., Garcia, A., Rodriguez, M., & Pelham, Jr., W.E. (2016). Early intervention for children with behavior problems in summer settings: Results from a pilot evaluation in Head Start preschools. *Journal of Early Intervention*, *38*(2), 92-117. https://doi.org/10.1177/1053815116645923
28. He, Y., Gewirtz, A. H., Lee, S., & August, G. (2018). Do parent preferences for child conduct problem interventions impact parenting outcomes? A pilot study in community children's mental health settings. *Journal of Marital and Family Therapy*, *44*(4), 716-729.

29. Hiller, R. M., Apetroaia, A., Clarke, K., Hughes, Z., Orchard, F., Parkinson, M., & Creswell, C. (2016). The effect of targeting tolerance of children's negative emotions among anxious parents of children with anxiety disorders: A pilot randomised controlled trial. *Journal of Anxiety Disorders*, *42*, 52-59.

30. Jones, D. J., Forehand, R., Cuellar, J., Parent, J., Honeycutt, A., Khavjou, O., Gonzalez, M., Anton, M., & Newey, G. A. (2014). Technology-enhanced program for child disruptive behavior disorders: Development and pilot randomized control trial. *Journal of Clinical Child & Adolescent Psycholgoy*, *43*(1), 88-101. https://doi.org/10.1080/15374416.2013.822308

31. Lanier, P., Kohl, P.L., Benz, J., Swinger, D., Moussette, P., & Drake, B. (2011). Parent-child interaction therapy in a community setting: Examining outcomes, attrition, and treatment setting. *Research on Social Work Practice*, *21*, 689-698.

32. Lau, A. S., Fung, J., Ho, L., Liu, L., & Gudino, O. (2011). Parent training with high-risk imigrant Chinese families: A pilot group randomized trial yielding practice-based evidence. *Behavior Therapy*, *42*(3), 413-426. https://doi.org/10.1016/j.beth.2010.11.001

33. Lebowitz, E. R., Omer, H., Hermes, H., & Scahill, L. (2014). Parent training for childhood anxiety disorders: the SPACE program. *Cognitive and Behavioral Practice*, *21*(4), 456-469.

34. Lebowitz, E. R., Marin, C., Martino, A., Shimshoni, Y., & Silverman, W. K. (2020). Parent-based treatment as efficacious as cognitive-behavioral therapy for childhood anxiety: A randomized noninferiority study of supportive parenting for anxious childhood emotions. *Journal of the American Academy of Child & Adolescent Psychiatry*, *59*(3), 362-372.

35. Love, S. M., Sanders, M. R., Turner, K. M.T., Maurange, M., Knott, T., Prinz, R., Metzler, C., & Ainsworth, A. T. (2016). Social media and gamification: Engaging vulnerable parents in an online evidence-based parenting program. *Child Abuse & Neglect*, *53*(2016), 95-107. https://doi.org/10.1016/j.chiabu.2015.10.031

36. Marker, C. D., Comer, J. S., Abramova, V., & Kendall, P. C. (2013). The reciprocal relationship between alliance and symptom improvement across the treatment of childhood anxiety. *Journal of Clinical Child & Adolescent Psychology*, *42*(1), 22-33.

37. Mautone, J. A., Marshall, S. A., Sharman, J., Eiraldi, R. B., Jawad, A. F., & Power, T. J. (2012). Development of a family-school intervention for young children with attention deficit hyperactivity disorder. *School Psychology Review*, *41*(4), 447.

38. McCabe, K., Yeh, M., Lau, A., & Argote, C. B. (2012). Parent-child interaction therapy for Mexican Americans: Results of a pilot randomized clinical trial at follow-up. *Behavior Therapy*, *43*(3), 606-618.

39. McDonald, R., Dodson, M. C., Rosenfield, D., & Jouriles, E. N. (2011). Effects of a parenting intervention on features of psychopathy in children. Journal of Abnormal Child Psychology, 39, 1013-1023. https://doi.org/10.1007/s10802-011-9512-8

40. McKay, M. M., Gopalan, G., Franco, L., Dean-Assael, K., Chacko, A., Jackson, J. M., & Fuss, A. (2011). A collaboratively designed child mental health service model: Multiple family groups for urban children with conduct difficulties. *Research on Social Work Practice*, *21*(6), 664-674. https://doi.org/10.1177/1049731511406740

41. McNaughton, D. B., Cowell, J. M., & Fogg, L. (2015). Efficacy of a Latino mother-child communication intervention in elementary schools. *The Journal of School Nursing*, *31*(2), 126-134. https://doi.org/10.1177/1059840514526997

42. Myers, K., Vander Stoep, A., & Lobdell, C. (2013). Feasibility of conducting a randomized controlled trial of telemental health with children diagnosed with attention-deficit/hyperactivity disorder in underserved

communities. *Journal of Child and Adolescent Psychopharmacology*, *23*(6), 372-378. https://doi.org/10.1089/cap.2013.0020

43. Niec, L. N., Barnett, M. L., Prewett, M. S., & Shanley Chatham, J. R. (2016). Group parent–child interaction therapy: A randomized control trial for the treatment of conduct problems in young children. *Journal of Consulting and Clinical Psychology*, *84*(8), 682-698

44. Nieter, L., Thornberry, T., & Brestan-Knight, E. (2013). The effectiveness of group parent-child interaction therapy with community families. *Journal of Child and Family Studies*, *22*, 490-501.

45. Pasalich, D. (2015). Feasibility of intensive parent–child interaction therapy (IPCIT): Results from an open trial. *Journal of Psychopathology and Behavioral Assessment*, *37*(1), 38-49. https://doi.org/10.1007/s10862-014-9435-0

46. Pasalich, D. S., Witkiewitz, K., McMahon, R. J., Pinderhughes, E. E., & The Conduct Problems Prevention Research Group. (2016). Indirect effects of the fast track intervention on conduct disorder symptoms and callous-unemotional traits: Distinct pathways involving discipline and warmth. *Journal of Abnormal Child Psychology*, *44*, 587-597. https://doi.org/10.1007/s10802-015-0059-y

47. Pears, K. C., Kim, H. K., Healey, C. V., Yoerger, K., & Fisher, P. A. (2015). Improving child self-regulation and parenting in families of pre-kindergarten children with developmental disabilities and behavioral difficulties. *Prevention Science*, *16*, 222-232.
48. Pfiffner, L. J., Kaiser, N. M., Curner, C., Zalecki, C., Rooney, M., Setty, P., & McBurnett, K. (2011). From clinic to school: Translating a collaborative school-home behavioral Intervention for ADHD. *School Mental Health, 3*, 127-142. https://doi.org/10.1007/s12310-01-9059-4
49. Pfiffner, L.J., Villodas, M., Kaiser, N., Rooney, M., & McBurnett, K. (2013). Educational outcomes of a collaborative school-home behavioral intervention for ADHD. *School Psychology Quarterly*, *28*(1), 25-36.
50. Pina, A. A., Zerr, A. A., Villalta, I. K., & Gonzales, N. A. (2012). Brief report: Indicated prevention and early intervention for childhood anxiety: A randomized trial with Caucasian and Hispanic/Latino youth. *Journal of Consulting and Clinical Psychology*, *80*(5), 940-946. https://doi.org/10.1037/a0029460
51. Rabbitt, S. M., Carrubba, E., Lecza, B., McWhinney, E., Pope, J., & Kazdin, A. E. (2016). Reducing therapist contact in parenting programs: evaluation of internet-based treatments for child conduct problems. *Journal of Child and Family Studies*, *25*(6), 2001-2020.

52. Rajwan, E., Chacko, A., Wymbs, B. T., & Wymbs, F. A. (2014). Evaluating clinically significant change in mother and child functioning: Comparison of traditional and enhanced behavioral parent training. *Journal of Abnormal Child Psychology*, *42*(8), 1407-1412.

53. Reuland, M. M., & Teachman, B. A. (2014). Interpretation bias modification for youth and their parents: A novel treatment for early adolescent social anxiety. *Journal of Anxiety Disorders*, *28(*8), 851-864.

54. Rooney, M., Hinshaw, S., McBurnett, K., & Pfiffner, L. (2016). Parent Adherence in Two Behavioral Treatment Strategies for the Predominantly Inattentive Presentation of ADHD. *Journal of Clinical Child & Adolescent Psychology*, *47*(1), S233-S241.

55. Santisteban, D.A., Czaja, S.J., Nair, S.N., Mena, M.P., & Tulloch, A.R. (2017). Computer informed and flexible family-based treatment for adolescents: A randomized clinical trial for at-risk racial/ethnic minority adolescents. *Behavior Therapy*, *48*, 474-489.

56. Sheridan, S. M., Bovaird, J. A., Glover, T. A., Garbacz, S. A., Witte, A., & Kwon, K. (2012). A randomized trial examining the effects of conjoint behavioral consultation and the mediating role of the parent-teacher relationship. *School Psychology Review*, *41*(1), 23-46.

57. Sheridan, S. M., Ryoo, J. H., Garbacz, S. A., Kunz, G. M., & Chumney, F. L. (2013). The efficacy of conjoint behavioral consultation on parents and children in the home setting: Results of a randomized controlled trial. *Journal of School Psychology*, *51*, 717-733. https://doi.org/10.16/j.jsp.2013.09.003

58. Sibley, M. H., Pelham, W. E., Derefinko, K. J., Kuriyan, A. B., Sanchez, F., & Graziano, P. A. (2013). A pilot trial of supporting teens' academic needs daily (STAND): a parent-adolescent collaborative intervention for ADHD. *Journal of Psychopathology and Behavioral Assessment*, *35*(4), 436-449.

59. Smith, A. M., Flannery-Schroeder, E. C., Gorman, K. S., & Cook, N. (2014). Parent cognitive-behavioral intervention for the treatment of childhood anxiety disorders: a pilot study. *Behaviour Research and Therapy*, *61*, 156-161.

60. Stein, G.L., Kulish, A.L., Williams, C.S., Mejia, Y.C., Prandoni, J.I., & Thomas, K.C. (2017). Latina/o parent activation in children's mental health treatment: The role of demographic and psychological factors. *Journal of Latina/o Psychology*, *5(*4), 290-305.

61. Stokes, J. O., Jent, J. F., Weinstein, A., Davis, E. M., Brown, T. M., Cruz, L., & Wavering, H. (2016). Does practice make perfect? The relationship between self-reported treatment homework completion and parental skill acquisition and child behaviors. *Behavior Therapy*, *47*(2016), 538-549. https://doi.org/10.1016/j.beth.2016.04.004

62. Tamm, L., Nakonezny, P. A., & Hughes, C. W. (2014). An open trial of a metacognitive executive function training for young children with ADHD. *Journal of Attention Disorders*, *18*(6), 551-559.

63. Vander Stoep, A., & Myers, K. (2013). Methodology for conducting the children's attention-deficit hyperactivity disorder telemental health treatment study in multiple underserved communities. *Clinical Trials*, *10*(6), 949-958.

64. Villodas, M. T., McBurnett, K., Kaiser, N., Rooney, M., & Pfiffner, L. J. (2014). Additive effects of parent adherence on social and behavioral outcomes of a collaborative school-home behavioral intervention for ADHD. *Child Psychiatry and Human Development*, *45*, 348-360. https://doi.org/10.1007/s10578-013-0405-7

65. Vujnovic, R., Fabiano, G. A., Pariseau, M. E., & Naylor, J. (2013). Parameters of adherence to a yearlong daily report card intervention for students with attention-deficit/hyperactivity disorder. *Journal of Educational and Psychological Consultation*, *23*(2), 140-163.

66. Walton, J. R., Mautone, J. A., Nissley-Tsiopinis, J., Blum, N. J., & Power, T. J. (2014). Correlates of treatment engagement in an ADHD primary care-based intervention for urban families. *The Journal of Behavioral Health Services & Research*, *41*, 539-547. https://doi.org/10.1007/s11414-013-9385-4

67. Weiss, B., Han, S., Harris, V., Catron, T., Ngo, V. K., Caron, A., Gallop, R., & Guth, C. (2013). An independent randomized clinical trial of multisystemic therapy with non-court-referred adolescents with serious conduct problems. *Journal of Consulting and Clinical Psychology*, *81*(6), 1027-1039. https://doi.org/10.1037/a0033928

68. Yasui, M., & Henry, D. B. (2014). Shared understanding as a gateway for treatment engagement: A preliminary study examining the effectiveness of the culturally enhanced video feedback engagement intervention. *Journal of Clinical Psychology, 70*(7), 658-672.
